# Atherogenic potential of microgravity hemodynamics in the carotid bifurcation: a numerical investigation

**DOI:** 10.1038/s41526-022-00223-6

**Published:** 2022-09-09

**Authors:** Philippe Sucosky, Varun Vinayak Kalaiarasan, Graham B. Quasebarth, Patricia Strack, Jason A. Shar

**Affiliations:** 1 Department of Mechanical Engineering, Kennesaw State University 840 Polytechnic Lane, Engineering Technology Center, MD #9075, Marietta, GA 30060 USA; 2 Department of Civil and Environmental Engineering, Kennesaw State University, 655 Arnston Drive, Civil and Environmental Engineering building, MD #9055, Marietta, GA 30060 USA

**Keywords:** Atherosclerosis, Biomedical engineering, Biophysics

## Abstract

Long-duration spaceflight poses multiple hazards to human health, including physiological changes associated with microgravity. The hemodynamic adaptations occurring upon entry into weightlessness have been associated with retrograde stagnant flow conditions and thromboembolic events in the venous vasculature but the impact of microgravity on cerebral arterial hemodynamics and function remains poorly understood. The objective of this study was to quantify the effects of microgravity on hemodynamics and wall shear stress (WSS) characteristics in 16 carotid bifurcation geometries reconstructed from ultrasonography images using computational fluid dynamics modeling. Microgravity resulted in a significant 21% increase in flow stasis index, a 22–23% decrease in WSS magnitude and a 16–26% increase in relative residence time in all bifurcation branches, while preserving WSS unidirectionality. In two anatomies, however, microgravity not only promoted flow stasis but also subjected the convex region of the external carotid arterial wall to a moderate increase in WSS bidirectionality, which contrasted with the population average trend. This study suggests that long-term exposure to microgravity has the potential to subject the vasculature to atheroprone hemodynamics and this effect is modulated by subject-specific anatomical features. The exploration of the biological impact of those microgravity-induced WSS aberrations is needed to better define the risk posed by long spaceflights on cardiovascular health.

## Introduction

Long-duration spaceflight poses multiple hazards to human health, including physiological changes associated with microgravity and increased exposure to radiation^1–4^. The acute cephalic fluid shift occurring upon entry into weightlessness is progressively compensated for by a reduction in blood volume, which effectively normalizes the blood pressure in the head and chest^1,3,4^. Those cardiovascular deconditioning events subject the arterial side of the circulation to substantial hemodynamic changes including a dramatic 16–18% reduction in mean flow rate in cerebral regions and a more moderate 2–6% reduction in mean flow rate in the lower limbs^5^.

The sustained exposure to such extensive alterations during long-term spaceflight combined with the known sensitivity of vascular endothelial cells to their hemodynamic environment raises some concerns regarding the possible impact of long-duration spaceflight missions on cardiovascular health. The first evidence of causality between long-term microgravity spaceflight and cardiovascular disease was suggested in a recent prospective study conducted on 11 astronauts participating in a long-duration spaceflight mission aboard the International Space Station^6^. The study, which aimed at quantifying jugular vein hemodynamics using ultrasound Doppler velocimetry, evidenced the existence of stagnant and retrograde flow conditions in six crew members, and the formation of an occlusive thrombus during spaceflight in the internal jugular vein of one more. Although those phenomenological observations did not establish causality between microgravity, flow stasis and thrombosis, they suggested that the blood flow alterations resulting from long-term exposure to weightlessness could be a newly discovered risk of spaceflight with potentially serious implications on cardiovascular health.

The suggested increased incidence of thrombosis under microgravity on the venous side^6^ alludes the possibility of long-term effects of microgravity on arterial occlusive disorders such as atherosclerosis. In fact, the formation of an atherosclerotic plaque on the arterial wall, which results from the accumulation of foam cells following lipid infiltration into the wall subendothelial layer, is driven initially by an increased endothelial permeability and an inflammation of the arterial wall^7–9^. Ground-based in vitro and clinical studies have demonstrated the occurrence of such atherogenic events in arterial regions experiencing low oscillatory flow and wall shear stress (WSS)^10,11^. Therefore, the reported reduction in cerebral arterial blood flow rate following long-term exposure to microgravity^5^ may promote the development of pro-atherogenic hemodynamics triggering the same biological cascades as those involved in atherogenesis on Earth. Testing this hypothesis requires the characterization of the fluid WSS alterations caused by long-term exposure to weightlessness on arterial wall regions particularly vulnerable to atherosclerosis. To address this need and account for inter-subject hemodynamic and anatomic variability, the objective of this study was to quantify computationally the impact of microgravity on the flow and WSS environments in 16 carotid bifurcation models reconstructed from ultrasonography images.

## Results

### Global flow structure, velocity field, and flow stasis

**Fig. 1:**
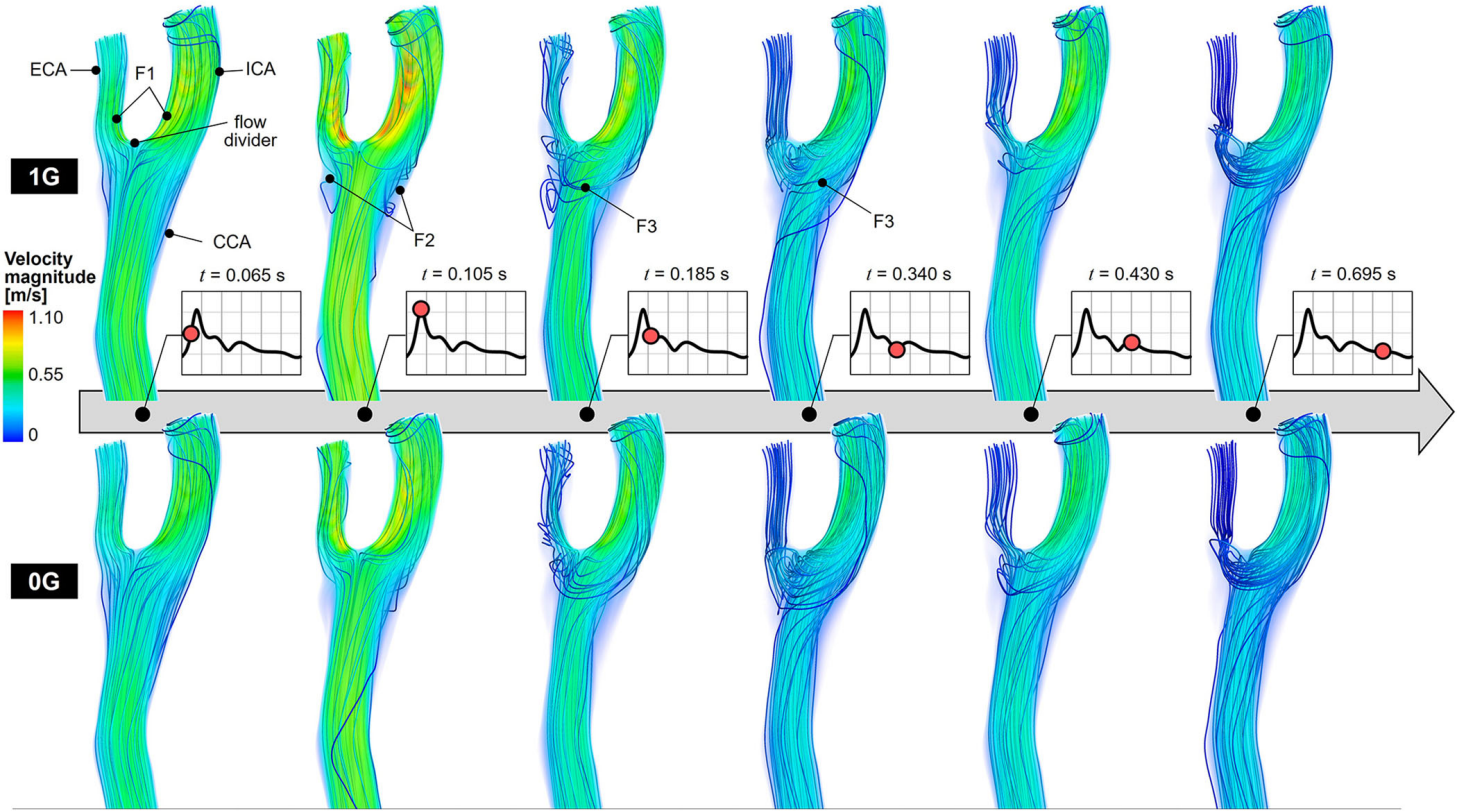
Comparison of flow velocity magnitude volume rendering and streamline field under 1G and 0G. The results are extracted from anatomy CB_3_ and are shown at six phases of the cardiac cycle. The inset indicates the current time point as a red dot on the CCA mass flow rate waveform.

**Fig. 2:**
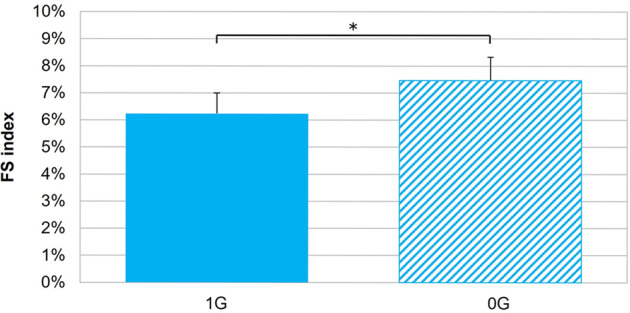
Comparison of the flow stasis (FS) index predicted under 1G and 0G (error bar: standard error; *n* = 16; *p < 0.05; paired Student *t*-test).

Snapshots of the flow velocity and streamline fields captured at 6 instants of time in anatomy CB_3_ under unit gravity (1G) and microgravity (0G) are shown in Fig. 1. An animation of the flow velocity magnitude and particle tracking captured in the same anatomy is provided in Supplementary Movie 1. The time history of the velocity fields suggests the weak dependence of the flow patterns on the gravity environment. During the acceleration phase (*t* = 0.065 s), the flow streamlines remain parallel to the wall and the impingement of the common carotid artery (CCA) jet near the flow divider gives rise to high flow velocities near the concavity of the external carotid artery (ECA) and internal carotid artery (ICA) (feature F1). At peak systole (*t* = 0.105 s), the separation of the high-momentum flow from the CCA wall upstream of the carotid sinus generates regions dominated by low velocities near the ECA and ICA convexity (feature F2). In those regions, the streamlines exhibit a high degree of curvature and flow reversal. During the deceleration phase (0.185 s ≤ *t* ≤ 0.340 s), the sustained redirection of the CCA jet toward the ICA and ECA branches gives rise to a secondary flow as evidenced by the circumferential alignment and wrapping of peripheral streamlines around the main axial flow upstream of the flow divider (feature F3). Late diastole (*t* ≥ 0.430 s) reveals the existence of sustained secondary flow patterns and decreasing flow velocity magnitudes. Any trace of secondary and reversed flows vanishes by the next acceleration phase. While most of those flow features are also observed under microgravity, 0G has two notable effects on the flow structure. First, it results in a marked reduction in flow velocity magnitude at all phases of the cardiac cycle and in all branches of the bifurcation. Second, it attenuates the streamline curvature observed under 1G at peak systole near the ECA and ICA convexity. The impact of microgravity on flow velocity is supported by the differences in flow stasis (FS) index predicted under 1G and 0G (Fig. 2). Regardless of the gravity environment, regions of flow stasis where the flow velocity was smaller than 5% of the 1G maximum peak-systolic velocity were relatively limited (<8% of the total fluid volume). However, 0G simulations resulted in a significant 21% increase in FS index relative to 1G (*p* < 0.05).

### Global WSS characteristics

**Fig. 3:**
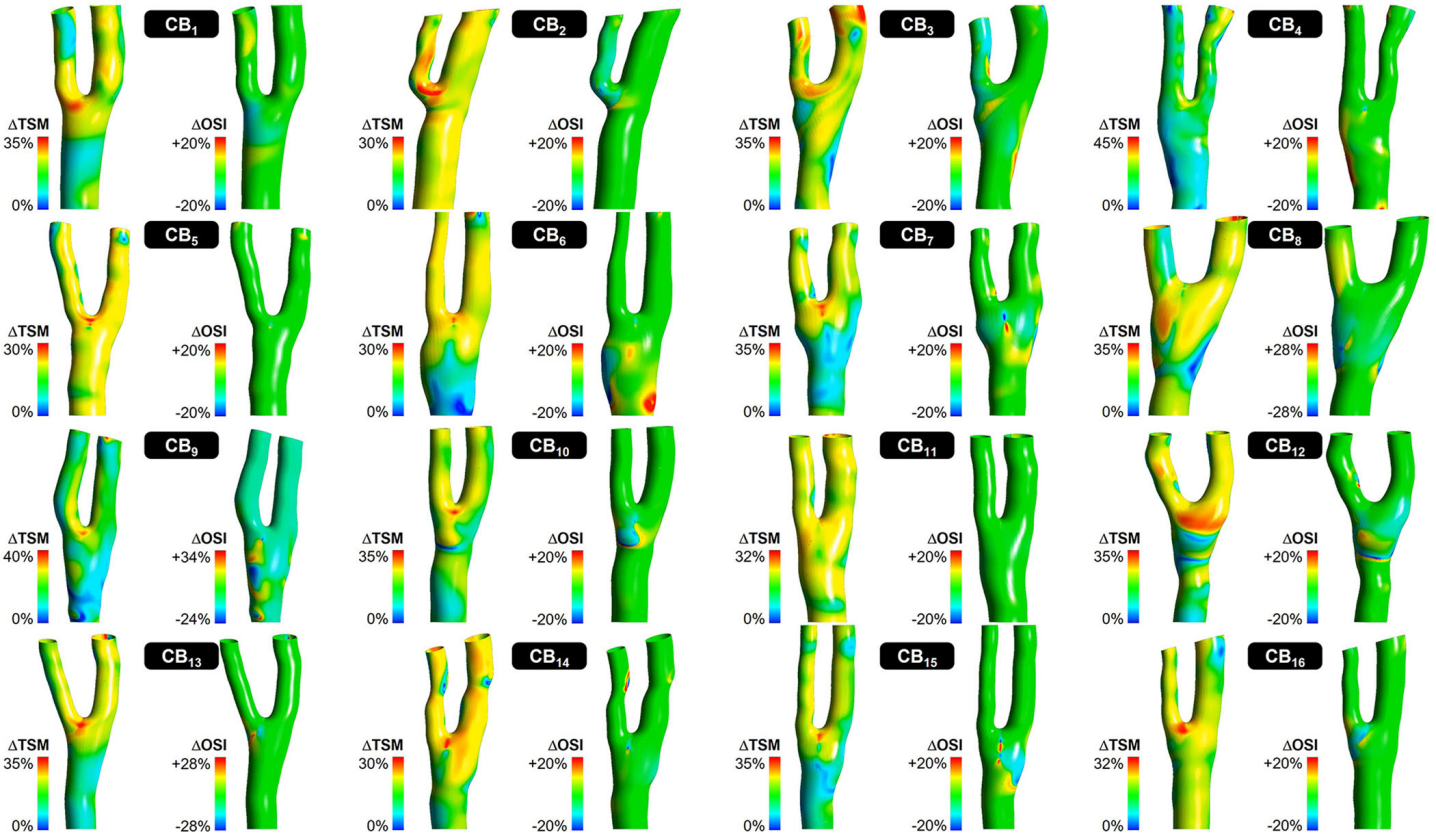
Changes in local WSS characteristics predicted between 1G and 0G in the 16 carotid bifurcation models. For each model, the left panel shows the local changes in TSM (ΔTSM), while the right panel shows the local changes in OSI (ΔOSI).

Changes in temporal shear magnitude (TSM) and oscillatory shear index (OSI) predicted in all 16 models under 0G relative to 1G (ΔTSM and ΔOSI, respectively) are shown in Fig. 3. A 360° animation of those fields is provided in Supplementary Movie 2. The analysis of the 0G WSS alterations reveals a high degree of spatial heterogeneity and inter-subject variability. 0G subjects all parts of the bifurcation to substantial changes in TSM, and the extent and location of those alterations differ dramatically between anatomies. However, three common features can be identified. First, the switch from 1G to 0G is systematically accompanied by a reduction in WSS magnitude (ΔTSM > 0). Second, while the exact wall location where the maximum change in TSM occurs varies between anatomies, it is systematically registered in the vicinity of the flow divider. Third, the magnitude of this change spans a relatively narrow range (30% < (ΔTSM)_max_ < 45%) across all models. Changes in OSI between 1G and 0G exhibit less spatial heterogeneity, and are only detected over small discrete wall regions. In contrast to the systematic reduction in TSM observed in all models, the effects of microgravity on WSS directionality are more complex with some wall regions experiencing increased WSS bidirectionality (ΔOSI < 0) and others exhibiting increased WSS unidirectionality (ΔOSI < 0). The locations of those regions are strongly dependent on the anatomical model.

### Regional WSS characteristics

**Fig. 4:**
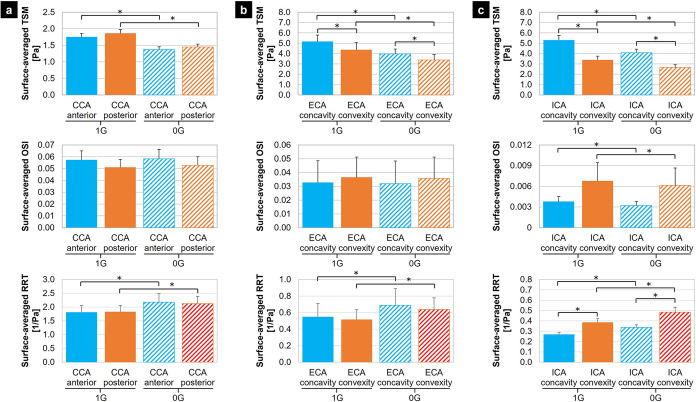
Regional WSS characteristics computed under 1G and 0G. Surface-averaged TSM, OSI and RRT extracted from: **a** the CCA, **b** the ECA, and **c** the ICA (error bar: standard error; *n* = 16; *p < 0.05; paired Student *t*-test).

The surface-averaged TSM, OSI and RRT predicted under 1G and 0G in six regions of the bifurcation and averaged over the 16 anatomical models are shown in Fig. 4. In the CCA, the WSS characteristics captured on the anterior and posterior walls exhibit nearly no side-specificity regardless of the gravity environment (less than 7% difference in TSM; less than 0.007-point difference in OSI; less than 3% difference in RRT). However, 0G subjects both the anterior and posterior CCA wall regions to significant changes in TSM (anterior CCA: 21.5% reduction vs. 1G; posterior CCA: 22.2% reduction vs. 1G; *p* < 0.05) and RRT (anterior CCA: 20.0% increase vs. 1G; posterior CCA: 16.2% increase vs. 1G; *p* < 0.05) without altering the OSI (less than 0.002-point increase vs. 1G). The results captured in the ECA convexity and concavity exhibit a different trend. In fact, while the RRT and OSI predictions in the wall convexity and concavity suggest their weak spatial dependence (less than 0.004-point difference in OSI; less than 8% difference in RRT), the simulations reveal the existence of a side-specific TSM environment, with the convexity exhibiting 15.3% and 14.6% smaller TSM than the concavity under 1G and 0G, respectively (*p* < 0.05). However, similarly to the trend observed in the CCA, 0G results in a significant reduction in TSM (up to 23.2% reduction vs. 1G; *p* < 0.05) and a significant increase in RRT (up to 25.1% increase vs. 1G; *p* < 0.05), with nearly no change in OSI (less than 0.001-point decrease vs. 1G). Lastly, the TSM predictions in the ICA exhibit the same side-specificity as in the ECA (36.0% and 35.0% lower convexity TSM vs. concavity TSM under 1G and 0G, respectively; *p* < 0.05), and the same overall reduction under 0G (23.1% and 21.8% reduction vs. 1G in the concavity and convexity, respectively; *p* < 0.05). Although a significant decrease in OSI is detected under 0G in the wall convexity and concavity (*p* < 0.05), those changes are limited (less than 0.0007-point difference vs. 1G) and only marginally reinforce the unidirectionality of the WSS environment in those regions (OSI < 0.007). In this bifurcation branch, the RRT becomes side-specific (44% difference between convexity and concavity RRT in both gravity conditions; *p* < 0.05) and exhibits the largest increase under 0G as compared to those observed in the two other branches (up to 25.6% increase vs. 1G; *p* < 0.05).

### Outlying data results

**Fig. 5:**
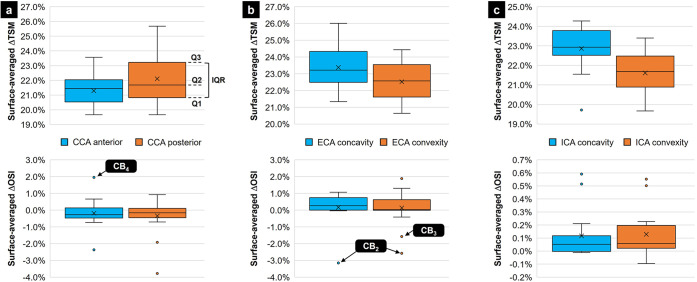
Box plots showing the analysis of the outlying regional WSS data. Surface-averaged ΔTSM and ΔOSI computed in **a** the CCA, **b** the ECA, and **c** the ICA (Q1: 1st quartile; Q2: 2nd quartile; Q3: 3rd quartile; ×: mean value; top and bottom whiskers: upper and lower limits calculated as *Q*3 + 1.5IQR and *Q*1 – 1.5IQR, respectively; text in bubble indicates outlying anatomies yielding trends opposite to the population-average trend).

The subject-specific analysis of the regional changes in TSM and OSI between 1G and 0G reveals a number of outlying data (Fig. 5). The relative change in TSM between 1G and 0G (ΔTSM) calculated in all 16 models reveals the existence of one outlier (marked as a point on the box plot) in the ICA concavity. This outlier generates a ΔTSM = +19.7%, which, despite being less substantial than the population-average change $\left( {\overline {{\Delta}{{{\mathrm{TSM}}}}} = + 22.9\% } \right)$, concurs with the general trend indicating a reduction in TSM under 0G. The calculation of the relative change in OSI between 1G and 0G (ΔOSI) results in a larger number of outlying data: two in the anterior CCA, two in the posterior CCA, one in the ECA concavity, three in the ECA convexity, two in the ICA concavity and two in the ICA convexity. Among those, three data points reveal changes in OSI which, in addition to being statistically different from the population-average value, are also opposite to the population-average trend. In anatomy CB_4_, 0G results in a decrease in OSI in the CCA anterior wall (ΔOSI = +2.0%), while the population-average value exhibits a marginal increase ($\overline {{\Delta}{{{\mathrm{OSI}}}}} = - 0.2\%$). In anatomies CB_2_ and CB_3_, 0G subjects the ECA wall to an increase in OSI (−3.2% < ΔOSI < −1.6%), which contrasts with the marginal decrease suggested by the population-average values ($\overline {{\Delta}{{{\mathrm{OSI}}}}} = + 0.16\%$ in the ECA concavity; $\overline {{\Delta}{{{\mathrm{OSI}}}}} = + 0.13\%$ in the ECA convexity).

## Discussion

The objective of this computational study was to predict the effects of 0G cardiovascular deconditioning on the hemodynamic and WSS environments in realistic carotid bifurcation anatomies. The analysis is not only the first to resolve the effects of 0G on the cerebral arterial vasculature but also to account for inter-subject anatomic and hemodynamic variability in the WSS characterization. The main contributions of the study are: (1) the demonstration of an increased degree of flow stasis and maintenance of WSS unidirectionality under 0G in most subjects; and (2) the suggestion that 0G may subject-specific carotid anatomies to a pro-atherogenic WSS environment marked by sub-physiologic magnitude and increased bidirectionality.

Both gravity conditions exhibited essentially similar flow structures that reveal the typical hemodynamic features of the carotid bifurcation^12–14^. The increase in streamline curvature and flow reversal predicted at peak systole in the carotid sinus opposite to the flow divider, as well as the formation of secondary flow patterns during the deceleration phase have been previously reported in computational and experimental studies^14–19^. Although time-averaged WSS levels have not been reported in different regions of the carotid bifurcation, the surface-averaged TSM predictions obtained in the CCA, ICA, and ECA (ICA: 43.6 dyn/cm^2^; ECA: 47.8 dyn/cm^2^; CCA: 18.1 dyn/cm^2^) are consistent with the range of TSM values reported in carotid fluid-structure interaction simulations (ICA and ECA: 0 < TSM < 64 dyn/cm^2^; CCA: 0 < TSM < 28 dyn/cm^2^)^20^. Lastly, the occurrence of the maximum change in TSM between 1G and 0G in the vicinity of the flow divider is also consistent with the regions where maximum WSS magnitudes have been reported in carotid bifurcation anatomies^20,21^.

The long-term effects of 0G were modeled via two mechanisms: (1) the removal of the gravitational acceleration, and (2) an 18% reduction in mass flow rate resulting from the physiological adaptation to weightlessness. While the concurrent implementation of these two mechanisms resulted in substantial hemodynamic alterations, the study did not isolate the respective impact of each mechanism. In an effort to address this gap, we ran another flow simulation in anatomy CB_1_ by maintaining the same inflow and outflow conditions as those prescribed in the 1G model while turning off the gravitational acceleration to isolate its contribution to 0G WSS alterations. The absence of gravitational acceleration only resulted in a marginal 0.006% decrease in TSM in the ICA concavity relative to the predictions obtained in the 1G model with gravity enabled. This result clearly demonstrates that inertial, viscous and pressure forces dominate the flow over the body force in the carotid bifurcation. It also indicates that the WSS alterations predicted under 0G are primarily the result of the long-term physiological adaptations to weightlessness.

Despite the use of a relatively small sample size (*n* = 16), the comparison of the population-average and subject-specific WSS characteristics evidenced contrasted results. Based on the population-average results, 0G subjected the carotid arterial wall to sub-physiologic WSS levels while maintaining an essentially unidirectional WSS, which translated in turn in an increasing degree of wall stress disturbance (as indicated by an increased RRT). In three anatomies however (CB_2_, CB_3_, and CB_4_), not only was the magnitude of the change in OSI statistically greater than the population average, the direction of this change was also opposite to the population average. Similar discrepancies have been described previously in a computational study comparing carotid bifurcation hemodynamics in an averaged model and subject-specific models^21^. It was shown that the carotid bifurcation WSS patterns were affected by both the specific anatomical features and mass flow rate boundary conditions of each individualized model. The key role played by inter-subject anatomical variability on carotid bifurcation hemodynamics has also been suggested in another numerical investigation aimed at isolating the impact of carotid sinus shape and branch angle on flow structure and WSS distribution^22^. Those anatomical features were shown to affect the size and location of the recirculation zone in the sinus as well as the duration of flow separation during one cycle. The results described in the present study support those previous findings and demonstrate their validity under 0G. Given the established significance of WSS magnitude and directionality characteristics in atherogenesis, the results also justify the need for subject-specific models in future investigations of causality between 0G hemodynamics and vascular physiology/disease.

The vulnerability of the carotid bifurcation to atherosclerotic lesion formation on Earth has been described as a result of the complex and disturbed flow patterns generated in this region of the vasculature^10,23–26^, some of which mimic the types of WSS alterations detected in this study under 0G. In all models, 0G hemodynamics increased the degree of flow stasis and subjected the arterial wall to sub-physiologic WSS levels and increased WSS disturbance. On Earth, low disturbed WSS and flow stasis have been identified as potential drivers of endothelial dysfunction and arterial remodeling. Intermediate WSS levels (10–25 dyn/cm^2^) are considered atheroprotective in healthy arteries and maintain a quiescent endothelial phenotype. In contrast, low or sub-physiologic WSS levels (<10 dyn/cm^2^) tend to switch endothelial cells from a quiescent phenotype to a more atherogenic phenotype marked by increased endothelial permeability and apoptosis, adhesion receptors expression, and matrix metalloproteinase expression^10,27–30^. Flow stasis and low WSS have also been associated with structural changes and degenerative processes in the arterial wall, which may facilitate aneurysm growth and trigger rupture^31^. In this context, the reduction in WSS magnitude predicted under 0G in all anatomies is expected to increase the vulnerability of the arterial wall to atherogenesis. In two anatomies, the decrease in WSS magnitude caused by microgravity was accompanied by an increase in WSS bidirectionality in the ECA. Low oscillatory WSS has been shown to result in the upregulation of atherogenic genes and the downregulation of endothelial nitric oxide synthase, which plays a protective role against plaque formation^10,11,32^. Therefore, the increased degree of flow stasis and bidirectionality caused by 0G in some anatomies are expected to exacerbate the atherogenic risk posed by flow stasis alone. It is interesting to note that the most atheroprone WSS conditions (sub-physiologic WSS levels and increased WSS bidirectionality) predicted under 0G were detected in only two models (13% of all anatomies). While the population size considered in this study is too limited to draw any firm conclusion, this predicted incidence of atheroprone hemodynamics is much lower than the prevalence of atherosclerosis reported in the general population on Earth (25.9%)^33^. The difference may be explained by the limited number of anatomical models considered in the study but also the absence of any other atherogenic factors such as hypercholesterolemia and hypertension, which is adequate when considering the astronauts population.

Long-term spaceflight missions pose a number of risks to cardiovascular health resulting from microgravity-induced alterations in blood flow and exposure to radiation. Consistent with the findings of the present study, stagnant and retrograde flow conditions have been reported in the jugular veins of six astronauts participating in long-duration spaceflight missions aboard the International Space Station^6^. While causality was not demonstrated, the same study evidenced the existence of thrombosis in the vein of one other astronaut participating in the same long-term mission, suggesting the possible adverse effects of long-term exposure to 0G on the venous vasculature. Space radiation from galactic cosmic rays and solar proton events also has the potential to negatively impact the cardiovascular system^34^. Animal and in vitro studies have shown that terrestrial radiation causes endothelial cell and vascular smooth-muscle cell dysfunction via reactive oxygen species pathways and that reactive oxygen species can prime cells to respond more adversely to a secondary insult^34–36^. In this context, exposure to both space radiation and microgravity-induced flow aberrations such as those captured in the present study may have synergistic or combinatorial thrombogenic and atheroprone effects on the vasculature. Unfortunately, testing this hypothesis is still hampered by: (1) the limited knowledge of the effects of 0G hemodynamics on the regional vascular WSS; (2) the difficulty to replicate the combined effects of space radiation and 0G hemodynamics on the ground; and (3) the challenge to subject vascular tissue/cells to the native time-varying WSS environment experienced in vivo. Although the computational results presented in this study provide compelling evidence for the increased risk posed by microgravity for carotid atherosclerosis, the causality between microgravity WSS and arterial biology remains to be established. One possible strategy could consist of exploring this causality in vitro on the ground by using one of the shear stress bioreactor devices developed in our laboratory^37–39^. Similarly to the approach implemented to isolate the effects of WSS on heart valve calcification and aortic dilation^40–44^, those devices could be used to subject carotid tissue to their native time-varying 1G and 0G WSS environments. Subsequent biological assays could be performed to assess the different biological pathways involved in the early stage of atherogenesis. The implementation of such devices along with other technologies enabling the replication of space radiation on the ground could be instrumental to the exploration of the risk of cardiovascular adaptations contributing to adverse mission performance and health outcomes.

## Methods

### Geometrical models

**Fig. 6:**
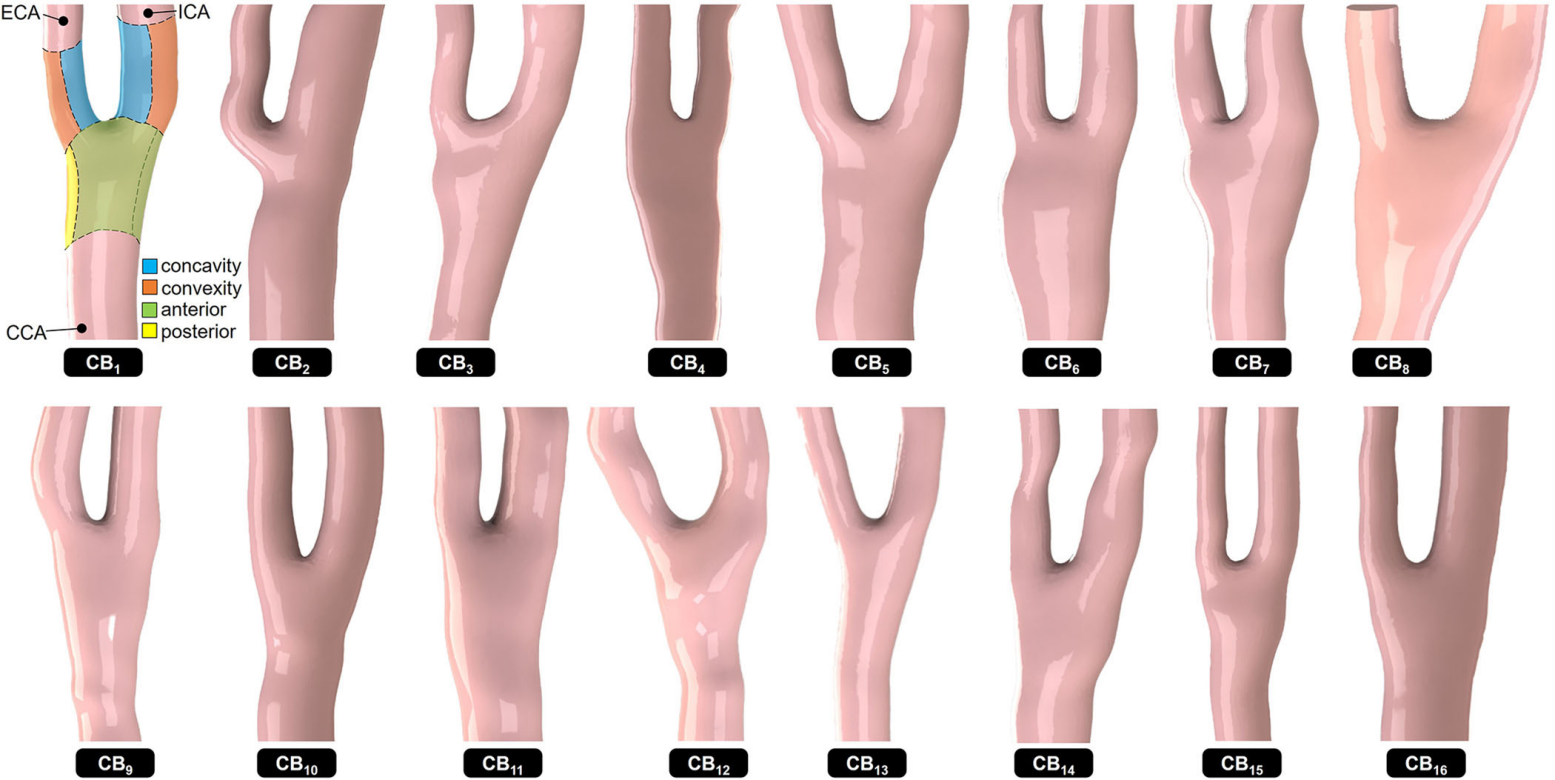
Description of the 16 reconstructed carotid bifurcation geometrical models. In each model, WSS characteristics were probed in six regions of interest (as shown in model CB_1_). Those include the posterior/anterior regions of the common carotid artery (CCA), and the concavity/convexity regions of the external carotid artery (ECA) and internal carotid artery (ICA).

The study was conducted on 16 carotid bifurcation anatomies obtained from healthy subjects between 18 and 30 years of age. The acquisition and reconstruction of those deidentified three-dimensional anatomies were performed previously by a different group using a novel high-resolution (pixel size: 0.0742 mm), two-dimensional ultrasound imaging technique^45^, which consisted of a MylabOne Vascular Ultrasound system (ESAOTE Europe B.V., Maastricht, NL) paired with a linear probe SL3323 and attached to a magnetic probe tracking device Curefab DS (Curefab Technologies GmbH, Munich, DE). The anatomical acquisition protocol was granted ethical approval from the ethics committee of the local hospital (Catharina Ziekenhuis, Eindhoven, NL) and all volunteers gave written informed consent. The resulting surface mesh files were converted into Parasolid files in the computer-assisted design (CAD) modeling software SolidWorks (Dassault Systèmes, Inc). The SpaceClaim ANSYS CAD module (ANSYS, Inc) was then used to identify the inlet and outlet sections of each model and generate the enclosed three-dimensional (3D) fluid domain. The resulting 16 carotid bifurcation geometries were identified as models CB_1_ through CB_16_ and included the CCA, ECA and ICA (Fig. 6). The measurements of the CCA, ICA, and ECA diameters in all 16 models yielded means ± standard deviations of 5.53 ± 0.52 mm, 4.39 ± 0.72 mm and 3.28 ± 0.48 mm, respectively (Table 1).

**Table 1:** Dimensional and 1G hemodynamic parameters in all 16 models.

Model	CCA diameter [mm]	ICA diameter [mm]	ECA diameter [mm]	CCA mean flow rate [mL/s]	CCA peak flow rate [mL/s]	CCA peak Reynolds number	CCA peak Womersley number
CB_1_	5.78	4.95	3.82	7.29	15.84	1057	8.50
CB_2_	6.65	6.20	3.05	9.75	21.20	1229	9.78
CB_3_	5.44	4.69	3.34	6.83	14.85	1053	8.00
CB_4_	5.98	4.63	3.68	7.97	17.32	1117	8.79
CB_5_	6.04	2.99	3.76	7.97	17.33	1106	8.88
CB_6_	5.00	3.60	2.11	5.44	11.83	912	7.35
CB_7_	5.58	4.57	3.18	7.04	15.31	1058	8.21
CB_8_	5.33	4.82	3.81	6.49	14.11	1021	7.84
CB_9_	5.52	3.96	3.43	6.66	14.48	1012	8.12
CB_10_	6.14	4.60	3.62	8.22	17.88	1123	9.03
CB_11_	5.65	4.71	3.29	6.96	15.13	1032	8.31
CB_12_	5.39	3.92	3.66	6.34	13.78	986	7.93
CB_13_	4.59	4.02	3.02	4.60	10.01	841	6.75
CB_14_	5.37	4.42	3.24	6.29	13.67	981	7.90
CB_15_	5.01	3.67	2.90	5.53	12.02	925	7.37
CB_16_	4.98	4.47	2.51	5.43	11.80	914	7.32

### Mesh generation

The mesh parameters used to discretize the fluid domain were determined following a mesh sensitivity analysis. One geometry (CB_7_) was imported into the computational fluid dynamics (CFD) software ANSYS Fluent and the fluid domain was discretized using 6 levels of refinement by varying the global cell size from 100 to 350 μm, which resulted in computational grids between 188,000 and 4,400,000 cells. The geometry was subjected to steady peak-systolic 1G flow conditions (CCA inlet: 15.31 mL/s; ICA outlet: 7.84 mL/s) and the total drag force (i.e., pressure and viscous drag) predicted on the wall of the ECA (surface extending from the carotid flow divider to the ECA outlet) was used as the convergence metric. The results indicated that a cell size of 150 μm generated an ECA drag force estimate that was 1% different from that predicted with the smallest cell size, while resulting in 64% fewer cells. Therefore, 150 μm was adopted as the cell size in all 16 geometries. Since the characterization of the WSS environment was a primary objective of this study, the mesh was refined locally through a mesh inflation strategy, which consisted of generating 8 radial inflation layers from the arterial wall (growth rate: 1.2). This meshing strategy resulted in computational grid sizes ranging from 1.5 to 3 million cells depending on the anatomical model.

### Modeling strategy

Blood was modeled as an incompressible fluid (density: *ρ* = 1060 kg/m^3^). The dependence of the blood dynamic viscosity *μ* on strain rate $\dot \gamma$ was accounted via implementation of a Carreau fluid model defined as

1 $$\mu = \mu _\infty + (\mu _0 - \mu _\infty )\left[ {1 + (\lambda \dot \gamma )^2} \right]^{\frac{{n - 1}}{2}}$$

with zero- and infinite-shear viscosities *μ*_0_ = 0.056 kg/m·s and *μ*_∞_ = 0.0035 kg/m·s, respectively, a time constant *λ* = 3.313 s, and a power-law index *n* = 0.3568, as reported previously^46^. Consistent with clinical and in vitro measurements, which reported moderate Reynolds numbers and relatively streamlined flows in carotid bifurcations of healthy subjects^47–49^, the flow was modeled as laminar. The transient flow equations consisted of the conservation of mass,

2 $$\nabla \cdot {{{\mathbf{V}}}} = 0$$

and the linear momentum balance,

3 $$\rho \left[ {\frac{{\partial {{{\boldsymbol{V}}}}}}{{\partial t}} + \left( {{{{\mathbf{V}}}} \cdot \nabla } \right){{{\boldsymbol{V}}}}} \right] = \nabla \cdot {{{\boldsymbol{\tau }}}} + {{{\boldsymbol{f}}}}$$

where **V** is the velocity vector, **τ** is the stress tensor, and **f** is the body force per unit mass (1G simulations: **f** = **g** = gravitational acceleration vector; 0G simulations: **f** = **0**). Those equations were solved using a finite-volume method and the pressure-based coupled solver of ANSYS Fluent. A second-order upwind scheme for spatial discretization and a second-order temporal discretization scheme were implemented. The convergence criterion for the continuity and momentum equations was set at 10^−6^. A physiologic heart rate of 68 beats per minute was simulated, resulting in a cardiac period of 0.88 s. This period was discretized into 176 time steps, each spanning 5 ms. The simulations were run for two cardiac cycles to eliminate start-up effects and all hemodynamic endpoints were extracted from the third period.

### Boundary conditions

**Fig. 7:**
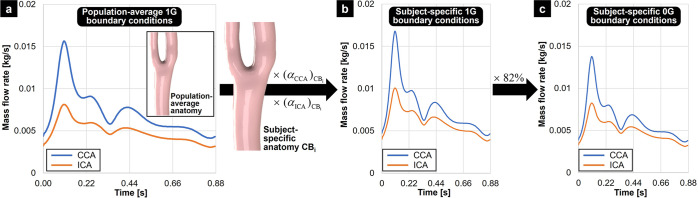
Strategy implemented to generate subject-specific 1G and 0G boundary conditions. **a** Reference 1G CCA and ICA mass flow rate conditions (adapted from Lee et al.^50^) were scaled based on the CCA and ICA diameters measured in each anatomical model to yield (**b**) subject-specific 1G CCA and ICA mass flow. **c** Subject-specific 0G mass flow rate conditions were then obtained by reducing the 1G conditions by 18%.

One critical aspect of the flow model was the prescription of realistic boundary conditions capturing: (1) the hemodynamic difference between unit gravity and microgravity, and (2) the hemodynamic variability between subjects. Owing to the absence of hemodynamic data associated with the 16 anatomies, the following protocol was followed to generate subject-specific boundary conditions (Fig. 7). First, published ICA and CCA mass flow rate waveforms corresponding to average ICA and CCA diameters^50^ and constructed from magnetic resonance imaging measurements in healthy subjects^51,52^ were considered representative of the 1G mean ICA and CCA mass flow rates in the 16 carotid bifurcation anatomies. The generation of subject-specific mass flow rate conditions relied on two previously validated assumptions: (1) the flow waveform shape is consistent across normal subjects^51^, and (2) the mean flow rate scales with the lumen cross-sectional area^53^. Therefore, for each anatomy (CB_i_), the reference CCA and ICA mass flow rates were adjusted by a scaling factor defined as the ratio of subject-to-reference CCA and ICA cross-sectional areas,

4 $$\left( {\alpha _{{{{\mathrm{CCA}}}}}} \right)_{{{{\mathrm{CB}}}}_{{{\mathrm{i}}}}} = \frac{{\left( {A_{{{{\mathrm{CCA}}}}}} \right)_{{{{\mathrm{CB}}}}_{{{\mathrm{i}}}}}}}{{\left( {A_{{{{\mathrm{CCA}}}}}} \right)_{{{{\mathrm{ref}}}}}}}$$

and

5 $$\left( {\alpha _{{{{\mathrm{ICA}}}}}} \right)_{{{{\mathrm{CB}}}}_{{{\mathrm{i}}}}} = \frac{{\left( {A_{{{{\mathrm{ICA}}}}}} \right)_{{{{\mathrm{CB}}}}_{{{\mathrm{i}}}}}}}{{\left( {A_{{{{\mathrm{ICA}}}}}} \right)_{{{{\mathrm{ref}}}}}}}$$

respectively, where (*A*_CCA_)_ref_ and (*A*_ICA_)_ref_ are the mean CCA and ICA cross-sectional areas of the 16 carotid bifurcation anatomies, and $\left( {A_{{{{\mathrm{CCA}}}}}} \right)_{{{{\mathrm{CB}}}}_{{{\mathrm{i}}}}}$ and $\left( {A_{{{{\mathrm{ICA}}}}}} \right)_{{{{\mathrm{CB}}}}_{{{\mathrm{i}}}}}$ are the CCA and ICA cross-sectional areas of the specific anatomical model CB_i_. Those scaling factors were applied to the reference mass flow rate waveforms to yield subject-specific CCA and ICA mass flow rates under 1G. Those boundary conditions resulted in a mean peak CCA Reynolds number of 1023 ± 97 and a mean peak CCA Womersley number of 8.13 ± 0.76 (Table 1). The generation of subject-specific boundary conditions representative of 0G hemodynamics was based on the results of a recently published one-dimensional lumped parameter model of the human circulation, which suggested an 18% reduction in mass flow rate through all carotid arteries under 0G^5^. Therefore, the subject-specific mass flow rate waveforms obtained under 1G were scaled by a factor of 82% to yield 0G mass flow rate conditions. The ECA outlet was assigned an “outflow” condition, which is suitable to model flow exits where the details of the flow velocity and pressure are not known prior to solving the flow problem.

### Hemodynamic characterization

Blood flow was characterized in terms of the global velocity field and instantaneous streamline fields. Flow stasis was also investigated and compared between 1G and 0G. For each carotid bifurcation anatomy, the peak-systolic velocity fields predicted under 1G and 0G were loaded and isovolumes were created to isolate regions of low flow velocities defined as

6 $$V \,<\, 0.05 \times V_{\max }$$

where *V* is the local flow velocity magnitude and *V*_max_ is the maximum 1G peak-systolic velocity magnitude. The volumes of those regions $\left( {{{{\boldsymbol{V}}}}_{{{{\mathrm{FS}}}}}} \right)$ calculated in 1G and 0G were then normalized by the volume of the fluid domain $\left( {{{{\boldsymbol{V}}}}_{{{{\mathrm{fluid}}}}}} \right)$ to yield a flow stasis index,

7 $${{{\mathrm{FS}}}} = \frac{{{{{\boldsymbol{V}}}}_{{{{\mathrm{FS}}}}}}}{{{{{\boldsymbol{V}}}}_{{{{\mathrm{fluid}}}}}}}$$

representing the volume fraction of the fluid domain dominated by low flow velocities.

The WSS environment on the arterial wall was quantified in terms of magnitude and directionality characteristics via the calculation of the temporal shear magnitude (TSM),

8 $${{{\mathrm{TSM}}}} = \frac{1}{T}{\int}_0^T {\left| \tau \right|} dt$$

and oscillatory shear index (OSI),

9 $${{{\mathrm{OSI}}}} = \frac{1}{2}\left[ {1 - \left( {\left| {{\int}_0^T {\tau \;dt} } \right|\left/{\int}_0^T\right. {\left| \tau \right|dt} } \right)} \right]$$

respectively. The TSM is a measure of the time-averaged WSS magnitude over one cardiac cycle, while the OSI characterizes the oscillatory nature of the WSS signal (OSI = 0: purely unidirectional; OSI = 0.5: purely oscillatory). In addition, regions of stress disturbance on the wall that are subjected to low WSS magnitude and high WSS oscillation were identified via the calculation of the relative residence time (RRT),

10 $${{{\mathrm{RRT}}}} = \frac{1}{{\left( {1 - 2 \times {{{\mathrm{OSI}}}}} \right){{{\mathrm{TSM}}}}}}$$

This metric is inversely proportional to the distance traveled during one cardiac cycle by a particle located near the wall^54^ and is a robust indicator of disturbed flow^55^. Global WSS alterations under 0G were quantified over the entire wall of each model by calculating the change in TSM relative to 1G and the change in OSI normalized by the maximum possible change ((ΔOSI)_max _= 0.5),

11 $${\Delta}{{{\mathrm{TSM}}}} = \frac{{({{{\mathrm{TSM}}}})_{{{{\mathrm{1G}}}}} - ({{{\mathrm{TSM}}}})_{{{{\mathrm{0G}}}}}}}{{({{{\mathrm{TSM}}}})_{{{{\mathrm{1G}}}}}}}$$

and

12 $${\Delta}{{{\mathrm{OSI}}}} = \frac{{({{{\mathrm{OSI}}}})_{{{{\mathrm{1G}}}}} - ({{{\mathrm{OSI}}}})_{{{{\mathrm{0G}}}}}}}{{({\Delta}{{{\mathrm{OSI}}}})_{\max }}}$$

respectively.

The WSS analysis was also performed regionally in the concavity and convexity of the ICA and ECA, and in the anterior and posterior regions of the CCA. Briefly, those regions were created in each anatomical model by first identifying the location of the flow divider, and identifying the regions extending two CCA diameters upstream, and two ECA/ICA diameters downstream. The resulting three regions were then split into halves (convexity and concavity in the ECA and ICA; posterior and anterior in the CCA) to yield six subject-specific wall regions (see Fig. 6). Surface-averaged ΔTSM and ΔOSI were calculated over each region in each model to enable a more consistent and localized comparison of the WSS metrics between models. Population average changes in TSM and OSI ($\overline {{\Delta}{{{\mathrm{TSM}}}}}$ and $\overline {{\Delta}{{{\mathrm{OSI}}}}}$) were also computed over each region to compare subject-specific results to global trends.

### Statistical analysis

All WSS data were processed using Microsoft Excel (Redmond, WA, USA) and analyzed using the open-source statistical software Jamovi version 2.0.0 (Jamovi Project, Sydney, Australia). In each region, WSS results were expressed in terms of the mean value over the 16 anatomies ± standard deviation. Statistical difference between 1G and 0G was investigated using two-tailed paired Student *t*-tests. A hypothesis test of the difference between population means and standard deviations was used. Statistical difference was considered at *p*-value < 0.05.

### Outlying data identification

Regional changes in TSM and OSI between 1G and 0G were also analyzed to identify potential outliers. ΔTSM and ΔOSI predicted in each anatomical region over the 16 models were used to calculate the first and third quartile values (*Q*1 and *Q*3, respectively) and to determine the interquartile range (IQR),

13 $${{{\mathrm{IQR}}}} = Q3 - Q1$$

Outliers were defined as values satisfying the following condition:

14 $$\left\{ {({\Delta}{{{\mathrm{TSM}}}}\;{{{\mathrm{or}}}}\;{\Delta}{{{\mathrm{OSI}}}})\, >\, Q3 + 1.5 \times {{{\mathrm{IQR}}}}} \right\}{{{\mathrm{or}}}}\left\{ {({\Delta}{\mathrm{TSM}}\;{{{\mathrm{or}}}}\;{\Delta}{\mathrm{OSI}})\, < \,Q1 - 1.5 \times {\mathrm{IQR}}} \right\}$$

### Limitations

A limiting aspect of the modeling strategy described in this study is the implementation of a rigid wall. Ultrasonography measurements conducted in 38 patients have shown an 11% increase in CCA diameter between diastole and systole^56^. The effects of wall compliance on the carotid WSS environment could be captured via the implementation of a fluid-structure interaction (FSI) modeling strategy. However, such a strategy would require wall mechanical properties describing the non-linear stress response to strain. While carotid FSI studies have approximated those properties as those measured in coronary arteries^56^, their possible alterations under long-term exposure to 0G remains largely unknown. Several computational studies have been conducted to quantify the error generated by the rigid-wall assumption in WSS predictions. One study aimed at quantifying blood flow metrics in a compliant carotid bifurcation reported a 25% reduction in WSS magnitude relative to its rigid-wall counterpart^9^. Another study conducted on a porcine right-coronary arterial tree reported the stronger impact of wall compliance on time-averaged WSS gradient (up to 16% reduction vs. rigid-wall model) than on WSS magnitude at bifurcation sites^10^. Therefore, the rigid wall implemented in our models may overestimate the near-wall velocity gradients and WSS magnitudes.

Additionally, the present models did not account for the vasolidation caused by 0G in the carotid arteries, which has been suggested by several studies^57–59^. Despite the large variability in wall distension reported across all studies and the lack of statistical significance, increases in CCA diameter measured during long-duration spaceflights range from 3 to 10%. A preliminary assessment of the effects of 0G-induced arterial distension on the WSS predictions was conducted in a dilated CB_1_ anatomy featuring an overall 4.7% increase in diameter relative to the original anatomy (consistent with previous measurements^59^). Vasodilation resulted in up to 23.7% reduction in peak-systolic WSS magnitude in the ICA. Although those results suggest that our models may underestimate the reduction in WSS caused by 0G, it is important to note that this effect might be compensated for by the increase in wall stiffness reported under 0G^60^. The assessment of the combined effects of 0G vasodilation and arterial stiffening would require the implementation of an FSI modeling strategy encapsulating all those effects in the same platform.

Other limiting factors are the simplified boundary conditions used to model the effects of microgravity and inter-subject variability. While the methodology used to generate subject-specific CCA and ICA mass flow rate conditions under 1G has been previously validated^50^, the 18% reduction in mass flow rate under 0G suggested by a recent computational 1D–0D multiscale model^5^ was applied uniformly across all anatomies. In addition, subject-specific boundary conditions were generated by scaling reference flow waveforms based on subject-specific anatomical dimensions. The availability of physiological data for each subject (e.g., heart rate, systolic/diastolic blood pressure, cardiac output) and the implementation of subject-specific lumped parameter models at the flow outlets would improve the modeling strategy. However, more experimental data describing the effects of the cephalic fluid shift and the reduction in blood volume following long-term adaptation to weightlessness on local cerebral blood flow is needed to better understand the long-term impact of microgravity on subject-specific carotid perfusion.

Lastly, the WSS characterization was performed in terms of established indicators such as TSM and OSI. Those metrics have been used historically to identify potential sites of vascular dysfunction^61^. However, those relatively simplistic metrics do not characterize the full complexity of the fluid shear stress at the wall nor can they identify all vascular regions where mechanotransduction may result in pathogenesis^62,63^. More sophisticated indicators focusing on specific WSS components (i.e., axial and transverse)^64^ or combining the local directionality and magnitude WSS characteristics^65^ have been proposed and could be explored in the context of microgravity hemodynamics. The analysis of the WSS topological skeleton and its key features (i.e., expansion and contraction regions, fixed points, and manifolds) is another strategy that has shown a lot of promise in identifying arterial wall regions exhibiting adverse biological responses to WSS^66,67^. The exploration of the carotid WSS aberrations caused by microgravity might benefit from a similar analysis.

### Reporting summary

Further information on research design is available in the Nature Research Reporting Summary linked to this article.

## Supplementary information

Supplementary Movie 1

Supplementary Movie 2

Reporting Summary Checklist

## Data Availability

The authors declare that the data supporting the findings of this study are available within the article and the supplementary material. All computational and statistical data in the article can be obtained from the corresponding author upon reasonable request.
